# Flow directionality, mountain barriers and functional traits determine diatom metacommunity structuring of high mountain streams

**DOI:** 10.1038/srep24711

**Published:** 2016-04-19

**Authors:** Xiaoyu Dong, Bin Li, Fengzhi He, Yuan Gu, Meiqin Sun, Haomiao Zhang, Lu Tan, Wen Xiao, Shuoran Liu, Qinghua Cai

**Affiliations:** 1 State Key Laboratory of Freshwater Ecology and Biotechnology, Institute of Hydrobiology, Chinese Academy of Sciences, Wuhan, China; 2 University of Chinese Academy of Sciences, Beijing, China; 3 Institute of Eastern-Himalaya Biodiversity Research, Dali University, Dali, China; 4 Collaborative Innovation Center for the Biodiversity in the Three Parallel Rivers of China, Dali, China

## Abstract

Stream metacommunities are structured by a combination of local (environmental filtering) and regional (dispersal) processes. The unique characters of high mountain streams could potentially determine metacommunity structuring, which is currently poorly understood. Aiming at understanding how these characters influenced metacommunity structuring, we explored the relative importance of local environmental conditions and various dispersal processes, including through geographical (overland), topographical (across mountain barriers) and network (along flow direction) pathways in shaping benthic diatom communities. From a trait perspective, diatoms were categorized into high-profile, low-profile and motile guild to examine the roles of functional traits. Our results indicated that both environmental filtering and dispersal processes influenced metacommunity structuring, with dispersal contributing more than environmental processes. Among the three pathways, stream corridors were primary pathway. Deconstructive analysis suggested different responses to environmental and spatial factors for each of three ecological guilds. However, regardless of traits, dispersal among streams was limited by mountain barriers, while dispersal along stream was promoted by rushing flow in high mountain stream. Our results highlighted that directional processes had prevailing effects on metacommunity structuring in high mountain streams. Flow directionality, mountain barriers and ecological guilds contributed to a better understanding of the roles that mountains played in structuring metacommunity.

Dispersal is one of the most important processes in biogeography, evolutionary biology and ecology. Dispersal affects the distribution of individuals in space, which in turn gives rise to genetic differentiation and changes the potential for local adaptation^1^. Traditionally, dispersal has been regarded as a population level character^2^; however, it also plays a significant role in community dynamics^3^,^4^. At the regional scale, dispersal shapes species diversity patterns^5^, and determines how much a regional pool contributes to species composition of local communities^6^. At the local scale, dispersal controls the loss (extinction, emigration) and gain (immigration, speciation) of individuals, regulates species coexistence through competition-colonization trade-off 7 and determines structure and function of local communities^8^. The theory of island biogeography and the neutral community models (NCM) are two examples which exemplify the effects of dispersal at the regional and local scales^9^,^10^.

Understanding the degree to which assemblages are dispersal limited could provide insights into the mechanism of community assembly and the maintenance of biodiversity^3^,^5^. On one hand, any factors which prevent organisms from moving between local habitats can be considered as barriers to dispersal^11^. The most common dispersal barriers are geographical barriers: dispersal limitation typically increased with increasing spatial distance between sites. Distance decay relationships (DDRs) describe this biogeographical phenomenon^12^. In addition, topographical and geomorphological factors (e.g. high mountains), hydrological factors, unsuited environmental conditions (e.g. deserts between sites), low connectivity of habitats, and artificial obstacles (e.g. dams for fish) also impede the dispersal of organisms^13^,^14^,^15^. On the other hand, ecological traits of species, such as dispersal ability or dispersal mode also determine the dispersal limitation^16^.

Community assembly is not only affected by dispersal occurring at regional scale, but also structured by local processes, such as environmental filtering and species interactions^17^,^18^. Metacommunity theory has provided an insightful view and a mechanism approach to study the dynamics of community patterns, species diversity and distribution at both local and regional scales^18^, which addresses ‘a set of local communities that are linked by dispersal of multiple potentially interacting species’^19^. Four conceptual paradigms have been put forward by Leibold *et al.*^17^ to describe metacommunities, depending on the relative importance of local and regional processes, termed as patch-dynamic (PD), species-sorting (SS), mass-effect (ME) and neutral-model (NM) paradigms. In the PD perspective, patches are identical and competition-colonization trade-off regulates metacommunity^20^. NM also assumes that patches are identical, but there is no competition–colonization trade-off (species are ecologically identical) and that stochastic speciation, extinction, immigration and migration determine species composition^21^. In contrast, SS and ME both assume patches and species are not identical. In SS, dispersal is not limited (moderate), so that species can track environmental gradients^22^, while in ME, the rate of dispersal is high enough to allow species survive in suboptimal patches via source-sink dynamics^18^,^23^. Another situation may arise if limiting dispersal (LD) occurs among patches, in that situation, the low-rate dispersal prevents species from being sorted to their optimal patches, and metacommunity presents a patch-dynamic paradigm^23^,^24^,^25^. Recently, many studies in this field have been published across a wide range of organisms and covering the terrestrial, marine and freshwater realms^26^,^27^. However, to our knowledge, few studies have been carried out in high mountain streams (but see^28^). Furthermore, most previous metacommunity studies were more concerned with geographic distance and many other dispersal limitation factors were largely ignored (but see^14^,^15^,^25^,^29^).

High mountain streams are often characterized by steep valleys, high elevational gradients and rushing flow. Geographical barriers occur frequently and across large areas^11^,^30^. For the organisms inhabiting high mountain streams, the steep slopes on both sides of the streams may constitute significant physical barriers to dispersal, even across small spatial extents^31^. For example, the Southern Rocky Mountains (mean altitude of *ca.* 3500 m) were proven to be a topographical barrier to the dispersal of a species of black fly (Simuliidae) (<800 km^2^)^32^. Similar results were shown by Šlechtová *et al.* for the fish in the Alps^33^. However, both studies mainly focused on the barrier effects at metapopulation level through molecular approaches, rather than from the metacommunity perspective. Furthermore, steep elevational gradients and high discharge can impose a significant directional dispersal along stream channels^34^,^35^. As a result, alternative dispersal pathways would be restricted compared with other systems, such as lakes or streams in flat areas^13^. Heino *et al.* who worked in River Oulankajoki basin which was characterized by considerable altitudinal differences suggested that high-rate dispersal would be important within streams^36^. Also a conceptual model focusing on the scale dependency of the mechanisms affecting metacommunities has supported the high-rate dispersal within streams and weaker dispersal among streams^37^. Steep elevational gradients also create more intense environmental gradients, such as temperature and conductivity^38^, which can influence the local processes more strongly than occurring in gentle slope areas. Accordingly, attempts to fully understand the mechanisms behind metacommunity structuring in high mountain streams must account for the intense environmental gradients and all kinds of dispersal limitation.

In this study, we investigated 63 sites in high mountain streams (elevation ranges from 1600 to 2900 m a.s.l.), across a small spatial extent in Southwestern China (<500 km^2^, Fig. 1). Benthic diatoms were selected to study metacommunity structuring in high mountain streams for the following reasons: (1) benthic diatoms are dominant primary producers, and one of the most important and ubiquitous assemblages in streams, however, to our knowledge, metacommunity study on benthic diatoms is still scarce up to now, especially those considering the dispersal ability or some other ecological traits when comparing with macroinvertebrates^11^,^39^ (but see^40^,^41^); (2) diatoms are widely regarded as ubiquitously distributed organisms^42^, but recent studies suggested that dispersal limitation also played an important role in structuring benthic diatom metacommunities in streams at a large scale^30^,^39^,^43^; (3) whether dispersal limitation occurs in high mountains at a small scale remains unknown; and (4) it is relatively easy to study the traits related to dispersal and habitat affinities of benthic diatoms^44^.

The aim of our study was to understand diatom metacommunity structuring in high mountain streams. To cover the unique characters of high mountain streams, geographical distance, topographic distance and network distance along flow direction were applied to model all pathways of dispersal (Fig. 2). Relative contributions of these three dispersal processes were then assessed to understand how high mountains restricted dispersal processes. Afterwards, we analyzed the relative influences of environmental vs. dispersal processes to explore the mechanism of community assembly. For a better understanding, benthic diatoms were categorized into three ecological guilds (namely high-profile, low-profile and motile guild^44^) to further examine the roles of functional traits. We proposed three hypotheses based on the unique characters of high mountain streams and species traits of benthic diatoms:

H1: Owing to steep slopes and high discharge, dispersal among streams will be limited, downstream along streams will be promoted.

H2: Steep elevational gradients will create intense environmental gradients in high mountain streams, giving rise to strong environmental effects.

H3: Ecological guilds are expected to respond to environmental and spatial factors differently: all guilds are expected to have significant relationships with environmental factors. While weak dispersers (the low-profile guild) will show strong spatial structure due to dispersal limitation, while strong dispersers (the high-profile and motile guild) will not.

## Results

### Community composition patterns

Across all 63 sites in our study area, a total of 149 diatom species belonging to 34 genera were identified. The number of species assigned to the high-profile, low-profile and motile guild were 49, 68 and 32 respectively. There were three dominant species in our study: *Achnanthidium rivulare* Potapova & Ponader, *Achnanthidium minutissimum* (Kützing) Czarnecki, and *Achnanthidium minutissimum* f. *inconspicuum* (Østrup) Compère & Riaux-Gobin, with relative abundance of 29.7%, 21.4%, and 13.2%, all belonging to the low-profile guild. The low-profile guild outweighed the other two guilds, contributing 45.6% of total richness and 95.3% of total abundance. Details on the species of each ecological guild were described in Supplementary Tables S1, S4 and S5.

Benthic diatom communities among the eight streams showed significant differences (adonis statistic R^2^ = 0.319, *P* = 0.001; ANOSIM statistic R = 0.24, *P* = 0.001; A = 0.157, *P* = 0.001 based on MRPP). Communities on the eastern slope were dissimilar from those on the western slope (adonis statistic R^2^ = 0.041, *P* = 0.022; ANOSIM statistic R = 0.062, *P* = 0.022; A = 0.018, *P* = 0.008 based on MRPP). Communities in the five streams on the eastern slope (adonis statistic R^2^ = 0.386, *P* = 0.001; ANOSIM statistic R = 0.443, *P* = 0.001; A = 0.197, *P* = 0.001 based on MRPP) and in the three streams on the western slope (adonis statistic R^2^ = 0.184, *P* = 0.003; ANOSIM statistic R = 0.111, *P* = 0.05; A = 0.079, *P* = 0.003 based on MRPP) were all distinctive in their composition.

### Relative importance of environmental and spatial factors

Variation partitioning was firstly performed on environmental variables (Env) and geographical spatial variables (PCNM_G_) for diatom communities. Both sets of variables were significant and could explain 54% variation of diatom communities (Fig. 3a). The variation purely explained by Env and PCNM_G_ was 22% and 12% respectively, while the shared fraction was 21% (Fig. 3a). Env and topographic spatial variables (PCNM_T_) explained 51% of the community variation. Env alone explained much more than PCNM_T_ (31% and 9%), while both explained 11% community variation (Fig. 3b). Directional spatial variables (AEM) had a unique contribution of 22% to metacommunity structuring, and the total explained variation increased to 65%. Furthermore, the unique faction of Env (3%) turned to be marginally significant (Fig. 3c).

### Comparison of dispersal modes

All of the three sets of spatial variables (i.e. PCNM_G_, PCNM_T_ and AEM) could significantly explain variation of diatom communities (*P* = 0.001 for PCNM_G_, *P* = 0.019 for PCNM_T_, and *P* = 0.016 for AEM, Table 1). Among them, AEM contained the most important spatial variables, as it accounted for 22% of the unique fraction and 40% of the shared variance (Fig. 4a). When variation partitioning was performed on PCNM_G_ and PCNM_T_ only, both of them had unique contributions (Fig. 4b), while AEM shared almost all explained variation with PCNM_G_ and PCNM_T_ (Fig. 4c,d).

### Analysis of deconstructed matrices

Among the three ecological guilds, different responses to environmental and spatial variables were revealed. Similar to the whole community, the low-profile guild had significant relationships with the environmental and the spatial variables (Table 1). The directional variables (AEM) also influenced the low-profile guild, with 13% of unique and 53% of shared fraction of variation explained (Table 2). In contrast, the high-profile guild could only be explained by environmental and topographic variables, with a total explained variation of 20%. Local effects were different between the high-profile and low-profile guild: conductivity, built-up% and TOC were the most important environmental variables for the low-profile guild, while altitude, depth, and pH were for the high-profile guild. Environmental variables contributed only a little to variation in the motile guild (5%), with spatial variables showing non-significant effects (*P* = 0.184, 0.832 and 0.464 for PCNM_G_, PCNM_T_ and AEM, Table 1).

## Discussion

From the four conceptual metacommunity paradigms^17^, species-sorting (SS) and mass-effect (ME) are the most commonly tested and supported paradigms in natural communities, both of which assumed that environmentally heterogeneous habitats were linked via sufficient dispersal^18^,^45^. The SS paradigm is highly related to niche theory, in which dispersal is unlimited and the environmental gradients along which species sort is the determinant of species distribution. As a comparison, in the ME paradigm, the local environment condition still plays an important role in structuring communities, but due to high rate of dispersal, the regional processes also show significant effects^12^,^18^. In ME (equivalent to SS+ high dispersal or SS+ HD in Ng *et al.* and SS+ ME in Cottenie)^23^,^45^, dispersal is so high that individuals can inhabit in less suitable habitats through source-sink dynamics^17^, and sink communities will be spatially structured. Nevertheless, spatial significance may result from not only ME, but also limiting dispersal (LD, synonymous with SS+ LD in Ng *et al.*)^23^, which can result in spatial significance by clustering individuals with similar dispersal ability across the landscape.

In our study, environmental factors contributed 42% of community variations (Table 1), supporting hypothesis H2, i.e. that steep elevational gradients create intense environmental gradients in high mountain streams, giving rise to strong environmental effects. In order to test metacommunity paradigms and to compare with previous studies, we firstly performed variation partitioning on environmental and geographical factors (the spatial factors which were most commonly used in testing metacommunity paradigms in previous studies), and both environmental and geographical effects controlled benthic diatom metacommunity (Fig. 3a). We summarized most previous researches on benthic diatom metacommunities in lotic systems (see Supplementary Table S2), and found that the relative importance of environmental and spatial factors varied among them. Besides differences in characters of the study areas, the importance of these factors seemed to depend on the spatial extent of the study^11^,^39^,^46^. Generally, diatoms were always species-sorting (only environment variables had significant effects), and the importance of spatial effects increased with geographical distance as dispersal limitation^45^,^47^, which was in line with the conceptual model from Soininen^39^. At large scales, spatial effects might even overcome environmental effects^30^,^43^. In general, the paradigms of benthic diatom metacommunities at large scales were always marked by SS+ LD. Also, Heino *et al.*^11^ suggested that at small extents, mass effects would increase in importance with shorter distances. Nevertheless, besides geographical distances, the influences of topographical and hydrological barriers for dispersal haven’t been considered in previous studies on benthic diatoms. At our study extent (spatial extent: <500 km^2^, max. distance between sites *ca*. 30 km), spatial effects (geographical factor, 12%) also played a significant role in structuring the metacommunity. However, given the characters of high mountain areas, whether the spatial effects attributed to mass effects arising from fast flow along streams or limiting dispersal (LD) caused by mountain barriers even at a small scale should be further analyzed. As a result, we investigated dispersal mechanism by comparison of dispersal modes, i.e. across topographic distance or along with flow.

Kristiansen^48^ has reviewed the four main modes of algae dispersal, among which dispersal by flow was the most natural way. Overland dispersal could be airborne dispersal by wind, or occur by virtue of organisms from aquatic insects, water birds to water-living mammals, even by humans. Previous studies on benthic diatoms considered mostly geographical distances as spatial factors in metacommunity analyses (Table S2), but ignored other barriers to dispersal. In our study, we modeled the possible ways of diatom dispersal, and transformed them into different sets of spatial factors (geographical, topographical and flow directionality) to explain metacommunity structure. Geographical distance was one of spatial factors controlling benthic diatom communities at a small spatial extent (*P* = 0.001, Table 1), which was in agreement with the study in a stream in Laojun Mountain^49^. Topographic factor also had a significant relationship with communities (*P* = 0.019, Table 1), indicating that the mountains acted as barriers for dispersal, similar results were also shown by Kärnä *et al.*^15^. Through nonparametric tests, significant distinctness of community composition was shown between streams, supporting hypothesis H1 that mountains could limit the dispersal among streams. Furthermore, directionality played a key role in shaping the metacommunity, confirming H1 that downstream along streams would be promoted by steep slopes and high discharge. The directional factor (AEM) not only uniquely explained the largest fraction of variation, but also substantially shared all of the other explained fractions with environmental and other spatial factors (Table 2; Figs 3c and 4a,c,d). This suggested that ecological processes were almost constrained along the stream corridors with directional flow in our study area. In conclusion, the strong spatial patterns of the metacommunity structure could be mainly ascribed to the strong directionality of flow, which could be regarded as a manifestation of mountain barriers. Flow promoted high rate of asymmetric dispersal of benthic diatoms, and generated a ME paradigm. Similar ideas have been suggested by Heino *et al.*^36^ and Heino, Melo & Bini^37^. Moreover, in the full model, the environmental factors (Env) could not singly contribute a faction, indicating that not only the dispersal processes constrained within streams, but also environmental gradients shaped by directional processes. The unexplained fraction (35%) in the full model might be explained by biotic interaction and some other factors.

Compared to the study in the Dong River^41^, one of the few available studies on effects of directionality on diatoms in lotic systems, our result demonstrated much stronger directional effects (unique contribution of 22% in the Cangshan versus <10% for all seasons or function groups in the Dong River). The specific characteristics of the high mountains, such as steep slope, gave rise to stronger and more powerful directional spatial processes, consistent with the viewpoint of Adams *et al.*, Lowe *et al.* and Altermatt *et al.*^34^,^35^,^50^. A study on headwater macroinvertebrates of Sweden demonstrated a significant but less marked directional spatial process compared with our study^25^. Similar results were also illustrated by studies on fish larvae in a fluvial lake system^51^ and coastal Mediterranean polychaetes^26^. Our results and those from other publications mentioned here suggest that directional processes are crucial in structuring diatom metacommunities, especially in high mountain streams, and cannot be neglected in future researches.

The deconstructive analysis based on ecological traits allowed a better understanding of processes underlying community structure patterns^16^,^40^. The ecological guilds of diatoms could reflect not only the difference of dispersal ability, but also the environmental adaptability^44^. Our results based on species traits suggested that ecological guilds were structured by different dynamic paradigms, which partly supported our hypothesis H3, i.e. that ecological guilds would respond to environmental and spatial factors differently. The low-profile guild could resist high disturbance but not resource stress^44^, which explained why the low-profile guild prevailed in high mountain streams, which were often characterized by oligotrophy and high discharge. Considering the steep slopes and the high water velocities, the low-profile species were still strongly controlled by directional factor (AEM), leading to a mass-effect paradigm (i.e. species sorting + high dispersal, SS+ HD). When compared to the low-profile guild, the dispersal of the high-profile guild seemed easier either along stream or among streams with flying grazers, such as water birds or odonata. However, limited by hydrological conditions, the total abundance of the high-profile species (only accounted for 4%) was too low to generate mass effects along with stream flow (i.e. species sorting + moderate dispersal, SS). In addition, it showed dispersal limitation for topographical barriers. The environmental and topographical effects on the high-profile guild indicated a combination of species sorting within streams and limiting dispersal among streams (SS+ LD, Table 2). The motile guild had a significant relationship with environmental variables, but none with spatial variables. This was not surprising, the active moving processes of the motile guild were too weak to resist strong flow and grazers to select suitable microhabitats, and consequently, the guild was still imposed by flow to track environmental gradients. Similar to the high-profile guild, low abundance gave rise to a non-significant spatial structure (species sorting + moderate dispersal, SS). High mountains have shaped the benthic diatom assemblages dominated by the low-profile guild with overwhelming abundance, so that the traits of the low-profile guild played decisive roles in structuring metacommunity. In conclusion, metacommunity structuring differed among the three ecological guilds, and the relative abundance of each guild finally determined the paradigm of the whole metacommunity. However, the species composition varies depending on the study area. In Sweden, for example, headwater streams, lower altitude (mean altitude 321 m a.s.l.) and gentle slopes assembled communities dominated by the high-profile guild^40^, and thus the metacommunity were structuring as a SS paradigm, which supported our conclusion. The deconstructive analysis allowed to better understand how flow directionality and mountain barriers acted as determining factors in structuring metacommunity of streams: regardless of how strong the dispersal ability was, the dispersal within streams was unconstrained, while among streams was limited by mountains.

## Conclusions

The unique characters of high mountain streams determined metacommunity structuring through: (1) considerable environmental gradients; (2) dispersal limitation among streams by mountain barriers and dispersal facilitation along stream channels by rushing flow, regardless of dispersal ability; (3) directional processes overriding any other effects and generating a mass-effect (ME) paradigm; (4) finally, the domination of the low-profile guild, so that the way by which the low-profile guild structured profoundly shaped the paradigm of the whole metacommunity.

Our results highlighted that in diatoms, stream corridors are the primary dispersal pathway in high mountains, where rushing flow and steep slope facilitate dispersal of benthic organisms. For biodiversity conservation, maintaining the instream environmental flow and keeping various habitats among streams are of vital importance in high mountain streams. The unique characters of high mountain streams are needed to be better understood and considered in further research.

## Methods

### Study areas

**Figure 1:**
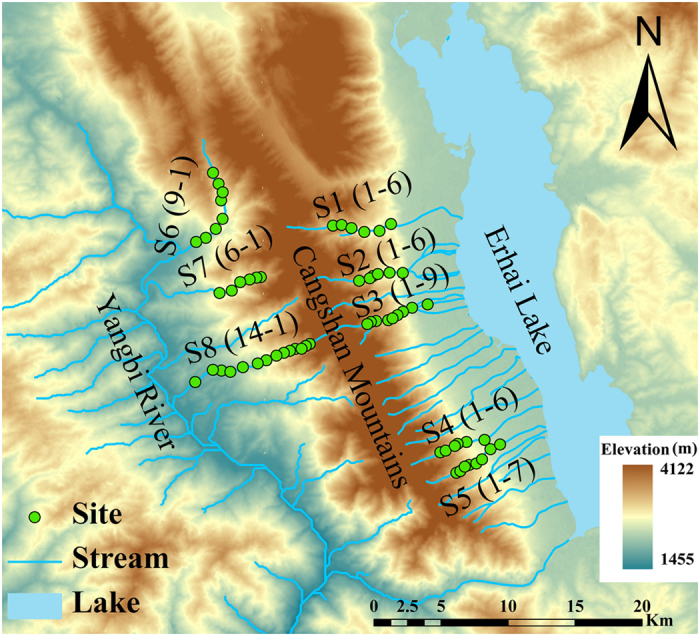
Map of the study area and the distribution of sampling sites in the Cangshan Erhai National Nature Reserve, Yunnan Province, Southwestern China. Green dots represent sampling sites (S1–1 represents the site most upstream in stream 1, and S1–6 represents the site most downstream in stream 1); blue lines and polygon depict the streams and the Erhai Lake. The map is based on a digital elevation model at 30 m resolution and created using ArcGIS 10.0 software (http://www.esri.com/software/arcgis).

**Figure 3:**
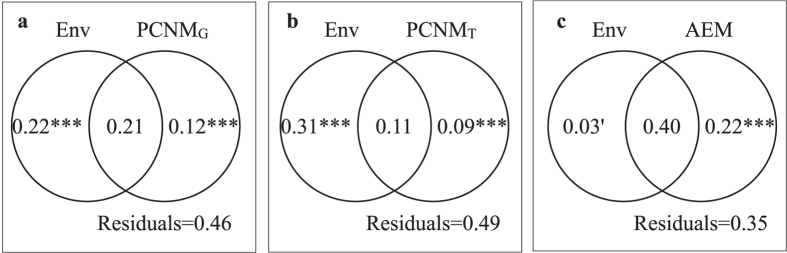
Venn-diagrams showing the results of variation partitioning performed on (**a**) Environmental model (Env) and Geographical model (PCNM_G_), (**b**) Environmental model (Env) and Topographic model (PCNM_T_), and (**c**) Environmental model (Env) and Directional spatial model (AEM) for benthic diatom metacommuntiy in the study area. Variation explained uniquely and jointly, and the unexplained fractions were shown as the number in each part of the figures (total variation = 1). The significance of each testable fraction was expressed as ^■^*P* < 0.1, ^*^P < 0.05, ^**^P < 0.01, ^***^P < 0.001.

**Figure 4:**
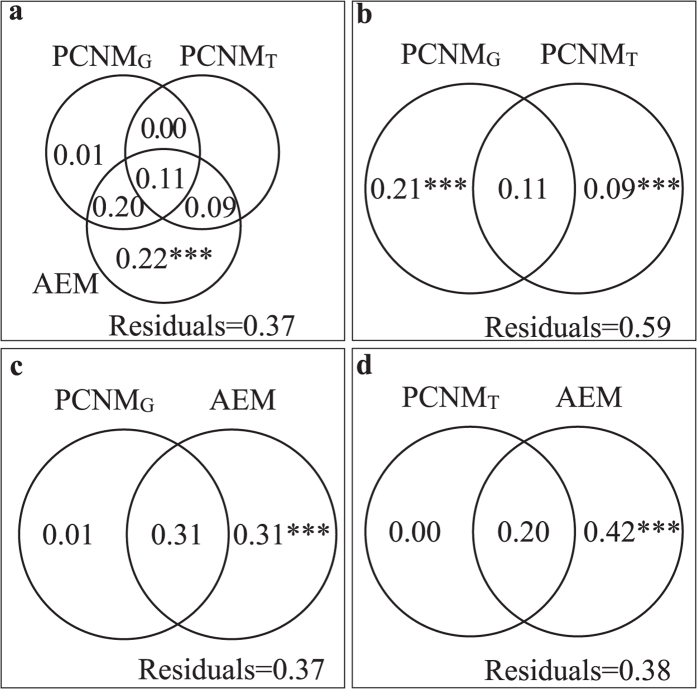
Venn-diagrams showing the results of variation partitioning performed on (**a**) Geographical model (PCNM_G_), Topographic model (PCNM_T_) and directional spatial model (AEM), (**b**) PCNM_G_ and PCNM_T_, (**c**) PCNM_G_ and AEM, (**d**) PCNM_T_ and AEM for benthic diatom metacommuntiy in the study area. Variation explained uniquely and jointly, and the unexplained fractions were shown as the number in each part of the figures (total variation = 1). The significance of each testable fraction was expressed as ^*^P < 0.05, ^**^P < 0.01, ^***^P < 0.001.

The study streams are located in the Cangshan Erhai National Nature Reserve, Yunnan Province, Southwestern China (25.64–25.85°N, 99.95–100.20°E, Fig. 1). The Cangshan Mountain, belonging to the Hengduan Mountains, is a part of the biodiversity hotspot “The Mountains of Southwest China” (Conservation International, http://www.conservation.org/Pages/default.aspx). The Cangshan Mountain summit reaches 4122 m a.s.l., and has 18 additional peaks all over 3000 m a.s.l., covering an area of about 950 km^2^. For these streams, the slope gradients range from 2.3 to 8.5%, and the length from 10 to 15 km. Land use is dominated by secondary forest (Table 3). A subtropical plateau monsoon climate prevails, characterized by two distinct seasons: wet (from May to October) and dry (from November to April of next year), where the rainfall in the wet season can account for up to 84% of the annual precipitation^52^. During November 2012, we investigated a total of 63 riffle sites from eight streams, five streams on the eastern slope and three on the western slope. Most sites were of difficult access to humans and in an almost pristine state.

### Diatom sampling and identification

At each site, 12 pebble-to-cobble-sized stones were collected randomly from riffle-run habitats in three typical sections within a 30 m stretch. Benthic diatoms attached on stone surface covered within a lid (with radius of 2.7 cm) were vigorously scrubbed by a nylon brush and flushed 3–4 times with distilled water. Three subsamples were subsequently pooled into one composite sample, and collected in a pre-defined volume container (350 ml) with 4% formalin.

Permanent diatom slides were prepared for diatoms identification after acid digestion. A minimum of 500 valves were counted at 1000 magnification under oil immersion (Olympus CX21, Japan). Diatoms were identified to the species level following the identification references of Krammer and Lange-Bertalot^53^ and Qi *et al.*^54^. The total number of diatoms were calculated and converted to abundance in unit area expressed as cells m^−2^.

### Species traits of diatom

Diatom species were assigned to three ecological guilds species-specifically based on their growth morphologies referring to Passy^44^, i.e. high-profile, low-profile and motile guild. The high-profile guild is composed of the species of tall stature beyond biofilm boundary, which are comparatively vulnerable to physical disturbances by flow and grazers, but relatively tolerant to resource limitation by extending beyond the boundary layer to exploit light and nutrients. The low-profile guild consists of species within boundary layers of biofilm, which are always resource-limited and much less prone to physical disturbance. The motile guild encompasses the relatively fast moving species, and therefore can actively select suitable microhabitats in some cases, comparatively free of both resource limitation and disturbance stress^44^. Based on above and considering environmental affinities, the low-profile guild should be subject to niche filtering because of limited resources; the high-profile guild is expected to be stressed by hydrological condition and grazers; while the motile guild is less sensitive to environmental filtering relatively. Concerning dispersal ability, the high-profile guild may be regarded as strong dispersers along stream flow or with flying grazers; as well as the motile species because of their active moving, while the low-profile guild is considered as weak dispersers. We also added some taxa recorded in this study but not mentioned by Passy^44^ to corresponding guilds based on their growth morphologies. Since the heterogeneous genera (i.e. *Navicula* and *Gomphonema*) just had one type of growth morphology in our study, the assignments of all species into three ecological guilds were based on their genera. Each genus assigned to three ecological guilds was shown on Supplementary Table S3.

### Environmental variables

At each site, conductivity (Cond), dissolved oxygen (DO), and pH were measured *in situ* with a multiparameter water quality meters (YSI Professional plus, US). Stream width was measured along 3 representative cross-transects. Depth and velocity were measured at 50 cm intervals across a transect using a digital water velocity meter (Global Water FP201, US). The mean values of stream width, depth and velocity were calculated for further analyses. Geographic coordinates and altitude were recorded using a hand-held GPS (Magellan 500E, US). A 350 ml water sample was collected simultaneously with diatom sampling and preserved by adding sulfuric acid to regulate pH < 2 in the field. Concentration of total phosphorus (T-P), Orthophosphate (PO_4_-P), total nitrogen (T-N), Nitrate nitrogen (NO_3_-N), Ammonium nitrogen (NH_4_-N), and dissolved silicon (SiO_2_-Si) were measured in the lab using a segmented flow analyzer (Skalar San^++^, Netherlands). Total organic carbon (TOC) was determined by using a TOC analyzer (Shimadzu TOC-V CPH, Japan).

Landsat data were downloaded from International Scientific & Technical Data Mirror Site, Computer Network Information Center, Chinese Academy of Sciences (http://www.gscloud.cn), and interpreted using ENVI (Version 4.1, ITT Visual Information Solutions Inc., US) to determine land cover/land use (LCLU) of our study area. The LCLU classification we used was a modification of Chinese National Standard “GB/T 21010-2007”. The LCLU categories included forest, grass, agriculture, built-up, water, and other (mainly barren land). ArcGIS software (Version 10.0, ESRI, US) was used to calculate the area of each land use category in the upstream watershed of each site^47^. The land-use areas were converted to proportions for use in future data analyses. A general description of the environmental variables used in our study is given in Table 3.

### Spatial variables and statistical analyses

#### Distance matrices

Three distance matrices were calculated: (a) a geographical distance matrix as Euclidean distance between each pair of sampling sites calculated using the *earth.dist* function in the package *fossil* in R (Version 3.0.0, R Development Core Team; package *fossil* Version 0.3.7); (b) a topographic distance matrix with pairwise landscape resistance distances to dispersal based on circuit theory^55^,^56^ generated with the program CIRCUITSCAPE (Version 4.0)^55^ using a digital elevation model (DEM, 30 m resolution, International Scientific & Technical Data Mirror Site, Computer Network Information Center, Chinese Academy of Sciences; http://www.gscloud.cn) and transformed into a curvature map from which resistance distances were calculated as the sum of the resistance of individual pixels in relation to topography (i.e. mountain barriers)^14^,^56^; and (c) a network distance matrix, calculated to model the least-cost dispersal route between two sites along the stream network, using the Network Analyst extension/OD Cost Matrix tool in ArcGIS 10.0.

#### Spatial variables

Three sets of spatial variables were extracted from the three distance matrices by eigenfunction-based spatial models in R. Principal Coordinates of Neighborhood Matrix (PCNM) analysis based on both geographical distance and topographic distance were used to model spatial variables representing geographical positions and dispersal across the mountains respectively^57^, through the *pcnm* function in package *vegan* (Version 2.0-10). We applied asymmetric eigenvector map (AEM) analysis, which was specifically designed to model directional patterns^58^, to generate spatial variables along directional flow. A site-by-edge binary matrix was constructed based on coordinates of the sites and the directional links (edges) among sites using the *build.binary* function in R package *AEM* (version 0.5-1/r188). Thereafter, the following weighting function was applied: weight = 1 − (*d/d*_*max*_)^2^, where *d* is the network distance between linked sites and *d*_*max*_ was the maximum distances among value *d*^59^, were assigned to each edge. Finally, the *aem* function in R package *AEM* was used to create eigenvectors. Details of AEM were described in Blanchet *et al.*^58^. The generated eigenvectors were used as spatial variables (i.e., PCNM_G_, PCNM_T_ and AEM components, PCNM_G_s, PCNM_T_s and AEMs) and hereafter referred to as PCNM_G_, PCNM_T_ and AEM.

#### Statistical analyses

All statistical analyses were conducted in R. Three nonparametric tests: permutational multivariate analysis of variance using distance matrices (adonis), analysis of similarity (ANOSIM) and multi-response permutation procedure (MRPP) were performed to test the differences of community composition and structure among the 8 streams in the study area, and between the eastern and western slope using the functions *adonis*, *anosim* and *mrpp* respectively in the package *vegan*.

To calculate the unique and shared effects of environmental and spatial variables, as well as how much community variation was explained by each set of the spatial variables, a variation partitioning analysis (partial RDA) was performed. First, a diatom abundance matrix (site-by-species) was Hellinger-transformed for further analyses^59^. Second, the environmental and spatial variables were prepared as site-by-variables matrices. Water chemistry and hydromorphological data were normalized by logarithmic or square root if necessary, and land-use data was transformed by a centred log ratio transformation (Table 3)^25^. Only the PCNM eigenvectors with positive eigenvalues were selected into spatial matrices. Third, the diatom matrix was analyzed by four explanatory matrices (one environmental and three spatial matrices). We performed a global test with redundancy analysis (RDA) using the *rda* function for each explanatory matrix and tested the significance using the *anova* function. Only if it was significant, a forward selection could be proceeded to get a parsimonious model with two stopping criteria: significance level and the adjusted coefficient of determination (R^2^adj) of the global model^60^. Forward selection was performed by the *forward.sel* function in the *packfor* package (version 0.0–8/r109). The selected variables were then used as explanatory variables for the following variation partitioning analysis by using *varpart* function. The significance of each testable fraction in variation partitioning analysis was obtained from the functions *rda* and *anova*. Similarly, the analyses mentioned above were implemented separately for each diatom ecological guild to test their different responses. The functions *varpart*, *rda* and *anova* are all found in the package *vegan*.

## Additional Information

**How to cite this article**: Dong, X. *et al.* Flow directionality, mountain barriers and functional traits determine diatom metacommunity structuring of high mountain streams. *Sci. Rep.* **6**, 24711; doi: 10.1038/srep24711 (2016).

## Supplementary Material

Supplementary Table S1-S4

Dataset1 (Table S5)

## Figures and Tables

**Figure 2:**
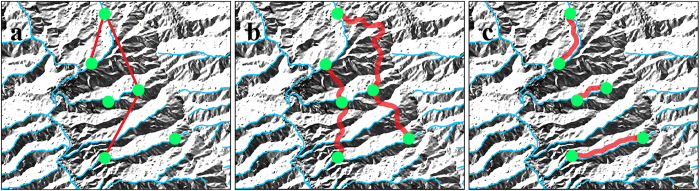
Hypothetical pathways of organisms’ dispersal among sites along or among streams in high mountains. (**a**) Geographical pathway (overland dispersal); (**b**) topographical pathway (across mountain barriers); (**c**) network pathway (along flow direction). The locations of communities are shown as green dots, streams are depicted by blue lines and red lines represent dispersal pathways between locations. The schematic diagrams were created with ArcGIS 10.0 (http://www.esri.com/software/arcgis) and modified with Adobe Photoshop CS6.

**Table 1:** Results of global test and forward selection for all taxa and each guild.

Group	Global test significance				Variables retained for variation partitioning from forward selection (AdjR^2^Cum)			
	*P*(Env)	*P* (PCNM_G_)	*P* (PCNM_T_)	*P* (AEM)	Env	PCNM_G_	PCNM_T_	AEM
All	0.001^***^	0.001***	0.019^*^	0.016^*^	Conductivity, Built-up%, Agriculture%, TOC, DO, Width, Grass%, PO_4_-P, Altitude, Barren land%, T-N, NO_3_-N (0.42)	PCNM_G_ 2, 6, 1, 25, 3, 13, 7 (0.32)	PCNM_T_ 5, 1, 3 (0.20)	AEM 4, 2, 8, 5, 1, 14, 10, 9, 3, 16, 15, 18, 6, 23, 11, 17, 26, 54, 19, 20, 13, 12, 60, 7, 27, 34, (0.61)
High	0.001^***^	0.146	0.026^*^	0.878	Altitude, Depth, pH, Built-up%, PO_4_-P, T-N (0.14)		PCNM_T_ 7, 5, 3, 43, 41, 1 (0.13)	
Low	0.001^***^	0.001^***^	0.013^*^	0.031^*^	Conductivity, Built-up%, TOC, DO, Agriculture%, Width, T-N, NO_3_-N (0.41)	PCNM_G_ 2, 6, 1, 25, 3, 13, 7, 21 (0.38)	PCNM_T_ 5, 1, 3 (0.23)	AEM 4, 2, 8, 5, 1, 14, 10, 3, 9, 15, 16, 18, 6, 23, 26, 54, 17, 11, 19, 20, 12, 13, 27, 28, 7, 60, 34 (0.68)
Motile	0.031^*^	0.184	0.832	0.464	Built-up%, NO_3_-N (0.05)			

Environmental model, Geographical model, Topographic model and Directional spatial model were expressed as Env, PCNM_G_, PCNM_T_ and AEM. *P* (Env), *P* (PCNM_G_), *P* (PCNM_T_) and *P* (AEM) give the significance of global tests (i.e. using all variables in each model). Only when global tests were significant, forward selections could be proceeded to get parsimonious models. The final retained variables are shown in the order in which they were selected in the forward selection procedure, with the AdjR^2^Cum of all retained variables in the following parentheses. The variables of spatial model were indicated as numbers, where small numbers represented broad-scales patterns and large numbers represented fine-scales. High, low and motile represented the high-profile, low-profile and motile guild, respectively. Significance was expressed as *P < 0.05, **P < 0.01, ***P < 0.001.

**Table 2:** Fractions of variation partitioning for all taxa and each guild.

	a	b	c	d	e	f	g	h	i	j	k	l	m	n	o	p
All	0.02	0.01	—	0.09^**^	—	0.003	0.01	0.12	0.03	0.03	0.17	—	0.06	0.1	0.04	0.35
High	0.05^**^	—	0.06^***^	—	—	—	0.09	—	—	—	—	—	—	—	—	0.80
Low	0.02^■^	0.07^**^	—	0.13^***^	—	—	0.004	0.11	0.07	0.03	0.14	0.005	0.04	0.07	0.08	0.25
Motile	0.05^*^															0.95

Environmental model, Geographical model, Topographic model and Directional spatial model were expressed as Env, PCNM_G_, PCNM_T_ and AEM. [a] unique environmental fraction; [b] unique PCNM_G_ fraction; [c] unique PCNM_T_ fraction; [d] unique AEM fraction; [e] shared fraction of Env and PCNM_G_; [f] shared fraction of PCNM_G_ and PCNM_T_; [g] shared fraction of Env and PCNM_T_; [h] shared fraction of Env and AEM; [i] shared fraction of PCNM_G_ and AEM; [j] shared fraction of PCNM_T_ and AEM; [k] shared fraction of Env, PCNM_G_ and AEM; [l] shared fraction of Env, PCNM_G_ and PCNM_T_; [m] shared fraction of PCNM_G_, PCNM_T_ and AEM; [n] shared fraction of Env, PCNM_T_ and AEM; [o] shared fraction of the four models; [p] unexplained fraction. High, low and motile represented the high-profile, low-profile and motile guild, respectively. Values <0 and no data were shown as —. Significance of each testable fraction (a–d) was expressed as ^■^*P* < 0.1, ^*^P < 0.05, ^**^P < 0.01, ^***^P < 0.001.

**Table 3:** Summary of environmental variables across 63 sites in the study area.

Environmental vaiables	Mean ± SD	Min-Max	Transformation
Altitude (m)	2309 ± 282.59	1623 ~ 2905	None
Conductivity (μs cm^−1^)	65.52 ± 30.82	16.80 ~ 151	None
DO (mg L^−1^)	7.88 ± 0.69	6.52 ~ 10.03	None
pH	7.96 ± 0.24	7.44 ~ 8.55	None
Stream width (m)	2.86 ± 1.69	0.36 ~ 10.70	Log
Depth (m)	0.20 ± 0.08	0.05 ~ 0.45	Log
Mean velocity (m s^−1^)	0.38 ± 0.22	0.06 ~ 1.27	Squ.root
T-N (mg L^−1^)	0.27 ± 0.27	0.02 ~ 1.29	Log
NO_3_-N (mg L^−1^)	0.22 ± 0.25	0 ~ 1.12	None
NH_4_-N (mg L^−1^)	0.02 ± 0.01	0.01 ~ 0.07	Log
T-P (mg L^−1^)	0.01 ± 0.004	0 ~ 0.03	None
PO_4_-P (mg L^−1^)	0.01 ± 0.004	0 ~ 0.02	Log
SiO_2_-Si (mg L^−1^)	4.31 ± 1.41	2 ~ 8.21	Squ.root
TOC (mg L^−1^)	0.38 ± 0.14	0.15 ~ 0.90	None
Forest %	81.93 ± 13.15	48.66 ~ 96.44	Centred log ratio
Grass %	9.05 ± 7.15	1.32 ~ 32.93	Centred log ratio
Agriculture %	0.52 ± 1.19	0 ~ 6.3	Centred log ratio
Built-up%	3.31 ± 2.40	0.13 ~ 9.32	Centred log ratio
Barren land %	5.19 ± 6.51	0.04 ~ 28.13	Centred log ratio

Transformations applied to meet normality assumptions are listed. None: none transformation; Log: logarithmic transformation; Squ.root: square root transformation; Centred log ratio: a transformation specially for compositional data.
