# Supplementary material for: Flow directionality, mountain barriers and functional traits determine diatom metacommunity structuring of high mountain streams
Source: Sci Rep. 2016 Apr 19;6:24711. doi: 10.1038/srep24711 (PMC4835781; doi:10.1038/srep24711)
Supplement: Supplementary Table S1-S4 [file srep24711-s1.doc]

**Supplementary:**

**Flow directionality, mountain barriers and functional traits determine diatom metacommunity structuring of high mountain streams**

Xiaoyu Dong, Bin Li, Fengzhi He, Yuan Gu, Meiqin Sun, Haomiao Zhang, Lu Tan, Wen Xiao, Shuoran Liu, Qinghua Cai^*^

**Supplementary Table S1** Statistical descriptions of richness and abundance for all taxa and each guild, and the relative abundance of dominant species for each group

| Group | Richness | Abundance (10^7^, cells m^-2^) | | |  | Dominant species (>10%) | |
| --- | --- | --- | --- | --- | --- | --- | --- |
|  |  | Total | Mean±SD | Min-Max |  | Species Relative abundance | |
| All | 149 | 4626.3 | 31.0±151.9 | 0.02~1372.1 |  | *Achnanthidium rivulare* | 29.7 % |
|  |  |  |  |  |  | *Achnanthidium minutissimum* | 21.4 % |
|  |  |  |  |  |  | *Achnanthes minutissima* var. *inconspicua* | 13.2 % |
| High | 49 | 183.1 | 3.74±8.65 | 0.02~51.6 |  | *Diatoma mesodon* | 28.2 % |
|  |  |  |  |  |  | *Gomphonema kobayasii* | 13.4 % |
|  |  |  |  |  |  | *Hannaea linearis* | 11.1 % |
| Low | 68 | 4411.1 | 64.9±221 | 0.05~1372.1 |  | *Achnanthidium rivulare* | 31.1 % |
|  |  |  |  |  |  | *Achnanthidium minutissimum* | 22.4 % |
|  |  |  |  |  |  | *Achnanthes minutissima* var. *inconspicua* | 13.8 % |
| Motile | 32 | 32.1 | 1±1.53 | 0.02~6.85 |  | *Navicula cryptotenella* | 21.3 % |
|  |  |  |  |  |  | *Navicula tenelloides* | 17.6 % |

Richness: The total number of taxa. Total abundance: the total number of cells in unit m^2^ area. Mean, SD, Min, Max abundance: the mean, standard deviation, minimum and maximum of abundance for taxa in each group. Relative abundance >10 % were regarded as dominant species.

**Supplementary Table S2** Summary of previous researches on effects of environmental and spatial factors on benthic diatoms in lotic systems

| **Habitat** | **Study area** | **Spatial scale** | **No. of sites** | **Elevation** | **Spatial variables** | **Total variation explained (TVE)** | **Spatial variables**  **(% TVE)** | **Environmental variables**  **(% TVE)** | **Shared**  **(% TVE)** | **Reference** |
| --- | --- | --- | --- | --- | --- | --- | --- | --- | --- | --- |
| Creek | White Creek (Wahington County), US | Extent: 16m^2^;  Interval of sites: 0.5 m | 81 |  | Trend surface analysis | 48 | 21 | 79 |  | Passy, 2001[^1^](#_ENREF_1) |
| River | Entire United States | Across level I ecoregions | 582 |  | Trend surface analysis | 11.9 | 28.3 | 57.7 | 14.0 | Potapova & Charles, 2002[^2^](#_ENREF_2) |
| River | Entire United States | Across level I ecoregions | 247 |  | Trend surface analysis | 18.9 | 29 | 57.9 | 13.1 | Potapova & Charles, 2002[^2^](#_ENREF_2) |
| River | US Ecoregion 5: Northern Forests | Within level I ecoregions,  Extent: 2,363,825 km^2^ | 26 | 334-678 m | Trend surface analysis | 83.7 | 30.9 | 43.6 | 25.5 | Potapova & Charles, 2002[^2^](#_ENREF_2) |
| River | US Ecoregion 6: North-western Forested Mountains | Within level I ecoregions  Extent: 1,788,950 km^2^ | 38 | 548-3408 m | Trend surface analysis | 74.6 | 29.7 | 51.4 | 18.9 | Potapova & Charles, 2002[^2^](#_ENREF_2) |
| River | US Ecoregion 8: Eastern Temperate Forests | Within level I ecoregions  Extent: 2,578,435 km^2^ | 306 | 9-1073 m | Trend surface analysis | 21.1 | 25.9 | 56.7 | 17.4 | Potapova & Charles, 2002[^2^](#_ENREF_2) |
| River | US Ecoregion 9: Great Plains | Within level I ecoregions  Extent: 3,543,875 km^2^ | 99 | 182-2606 m | Trend surface analysis | 41.1 | 24.2 | 54.6 | 21.2 | Potapova & Charles, 2002[^2^](#_ENREF_2) |
| River | US Ecoregion 10: North American Deserts | Within level I ecoregions  Extent: 2,027,460 km^2^ | 68 | 326-3230 m | Trend surface analysis | 50.4 | 23.4 | 67.2 | 9.4 | Potapova & Charles, 2002[^2^](#_ENREF_2) |
| River | US Ecoregion 8.1: Mixed Wood Plains | Within level II ecoregions | 44 | 93-454 m | Trend surface analysis | 56.8 | 16.7 | 72.7 | 10.6 | Potapova & Charles, 2002[^2^](#_ENREF_2) |
| River | US Ecoregion 8.2: Central USA Plains | Within level II ecoregions | 49 | 100-422 m | Trend surface analysis | 62.7 | 15.4 | 66.7 | 17.9 | Potapova & Charles, 2002[^2^](#_ENREF_2) |
| River | US Ecoregion 8.3: South-eastern USA Plains | Within level II ecoregions | 86 | 12-402 m | Trend surface analysis | 36.8 | 21.9 | 67.5 | 10.6 | Potapova & Charles, 2002[^2^](#_ENREF_2) |
| River | US Ecoregion 8.4: Ozark, Quachita Appalachian Forests | Within level II ecoregions | 96 | 130-1073 m | Trend surface analysis | 39.1 | 16.6 | 69.6 | 13.8 | Potapova & Charles, 2002[^2^](#_ENREF_2) |
| River | US Ecoregion 8.5: Mississipii alluvial and south-eastern coastal Plain | Within level II ecoregions | 31 | 9-111 m | Trend surface analysis | 72.8 | 21.8 | 61.9 | 16.3 | Potapova & Charles, 2002[^2^](#_ENREF_2) |
| Stream | Whole of Finland | Across level I ecoregions  Max distance: ca. 1,000 km | 197 |  | Trend surface analysis | 10.2 | 24 | 38 | 38 | Soininen, 2004[^3^](#_ENREF_3) |
| Stream | South boreal Ecoregion of Finland | Within level I ecoregions  Max distance: ca. 100 km | 92 | 80-360 m | Trend surface analysis | 13 | 20 | 55 | 25 | Soininen, 2004[^3^](#_ENREF_3) |
| Stream | Middle boreal Ecoregion of Finland | Within level I ecoregions  Max distance: ca. 100 km | 47 | 0-360 m | Trend surface analysis | 18.6 | 18 | 62 | 20 | Soininen, 2004[^3^](#_ENREF_3) |
| Stream | North boreal Ecoregion of Finland | Within level I ecoregions  Max distance: ca. 100 km | 33 | 150- 800 m | Trend surface analysis | 12.9 | 20 | 72 | 8 | Soininen, 2004[^3^](#_ENREF_3) |
| Stream | R. Vantaanjoki river, Finland | Watershed; Max distance: ca. 10-100 km | 21 |  | Trend surface analysis | 22.8 | 16 | 80 | 4 | Soininen, 2004[^3^](#_ENREF_3) |
| Stream | 15 stream riffles in three drainage systems, northern Finland | Max distance: 350 km | 45 |  | PCNM components | 12.4 | 28 | 58 | 14 | Soininen & Weckström, 2009[^4^](#_ENREF_4) |
| Stream | Ecoregions (no. 20 and 22) of Europe | Across most of Finland, Max distance: ca. 1,000 km | 223 | 4-539 m | PCNM components | 26 | 42.3 | 19.6 | 38.1 | Heino et al., 2010[^5^](#_ENREF_5) |
| Stream | Western Allegheny Plateau 58 streams, US | Extent: 30,828 km^2^ | 58 |  | Trend surface analysis | 38.9 | 27.8 | 56.8 | 15.4 | Smucker & Vis, 2010[^6^](#_ENREF_6) |
| Stream | Leading Creek watershed, US | Extent: 388 km^2^ | 18 |  | Trend surface analysis | 54.2 | 31 | 42.4 | 26.6 | Smucker & Vis, 2010[^6^](#_ENREF_6) |
| Stream | Shade River watershed, US | Extent: ca. 300 km^2^ | 21 |  | Trend surface analysis | 51.4 | 36.8 | 42.2 | 21 | Smucker & Vis, 2010[^6^](#_ENREF_6) |
| Stream | A stream in Laojun Mountain, China | Max distance: 33.5 km | 26 | 1820-4050 m | PCNM components | 75 | 16 | 6.7 | 77.3 | Wang et al., 2012 |
| Stream | South-central Sweden | Extent: ca. 14,000 km^2^ | 30 | 146-631 m | PCNM components | 38 | 13.2 | 50 | 36.8 | Göthe et al., 2013[^8^](#_ENREF_8) |
| River | Northern-central China | Max distance: 1,001.6 km | 28 |  | MEM & Trend surface analysis | 22 | 40.9 | 27.3 | 31.8 | Tang et al., 2013[^9^](#_ENREF_9) |
| Stream | Three catchments along Yangtze River | Max distance: ca. 1,500 km | 50 | 514-3144 m | Trend surface analysis | 64.7 | 30.6 | 8.3 | 61.1 | Wu et al., 2014[^10^](#_ENREF_10) |

**Supplementary Table S3** Taxa assignments to diatom ecological guilds in our study (adapted from Passy, 2007)[^11^](#_ENREF_11)

| Guild | Taxa |
| --- | --- |
| High-profile | *Aulacoseira, Diatoma, Didymosphenia, Eunotia, Fragilaria, Gomphonema, Hannaea, Melosira, Pinnularia, Pseudostaurosira, Synedra, Tetracyalus* |
| Low-profile | *Achnanthidium, Achnanthes, Amphora, Cocconeis, Cyclotella, Cymbella, Encyonema, Halamphora, Karayevia, Meridion, Planothidium, Platessa, Psammothidium, Reimeria, Rhoicosphenia* |
| Motile | *Denticula, Epithemia, Frustulia, Navicula, Nitzschia, Stauroneis, Surirella,* |

**Supplementary Table S4** Total abundance of each guild at each site in our study (unit: cells per m ^2^)

| Site | High-profile guild | Low-profile guild | Motile guild |
| --- | --- | --- | --- |
| S1-1 | 1.01E+07 | 3.63E+08 | 2.34E+06 |
| S1-2 | 5.61E+07 | 7.49E+08 | 7.79E+06 |
| S1-3 | 3.97E+07 | 4.83E+08 | 3.05E+06 |
| S1-4 | 3.38E+06 | 5.39E+07 | 5.19E+05 |
| S1-5 | 7.79E+05 | 5.53E+07 | 0 |
| S1-6 | 5.19E+05 | 6.02E+07 | 1.04E+06 |
| S2-1 | 2.34E+06 | 3.52E+08 | 0 |
| S2-2 | 3.12E+06 | 5.23E+08 | 7.79E+05 |
| S2-3 | 9.35E+06 | 5.11E+08 | 0 |
| S2-4 | 3.56E+06 | 2.04E+08 | 0 |
| S2-5 | 7.79E+05 | 5.84E+07 | 5.84E+05 |
| S2-6 | 6.23E+05 | 1.50E+07 | 3.12E+05 |
| S3-1 | 9.35E+06 | 2.47E+08 | 5.19E+05 |
| S3-2 | 9.35E+06 | 2.64E+08 | 0 |
| S3-3 | 1.40E+07 | 7.35E+08 | 0 |
| S3-4 | 1.06E+07 | 2.20E+08 | 0 |
| S3-5 | 2.83E+07 | 6.15E+08 | 4.25E+06 |
| S3-6 | 1.66E+07 | 5.63E+08 | 2.08E+06 |
| S3-7 | 2.28E+07 | 4.84E+08 | 6.23E+06 |
| S3-8 | 7.79E+06 | 7.36E+08 | 2.60E+06 |
| S3-9 | 8.57E+06 | 4.28E+07 | 1.56E+05 |
| S4-1 | 7.79E+06 | 3.63E+08 | 1.73E+06 |
| S4-2 | 5.71E+06 | 1.82E+08 | 1.04E+06 |
| S4-3 | 5.19E+06 | 5.22E+08 | 0 |
| S4-4 | 1.38E+07 | 1.00E+09 | 1.73E+06 |
| S4-5 | 9.00E+06 | 1.32E+08 | 0 |
| S4-6 | 5.19E+07 | 4.27E+08 | 0 |
| S5-1 | 6.31E+07 | 1.65E+09 | 4.21E+06 |
| S5-2 | 2.31E+07 | 1.15E+09 | 6.31E+06 |
| S5-3 | 7.54E+06 | 1.45E+09 | 5.02E+06 |
| S5-4 | 1.22E+08 | 3.34E+09 | 4.21E+07 |
| S5-5 | 8.41E+06 | 2.40E+09 | 4.21E+06 |
| S5-6 | 3.79E+07 | 1.69E+09 | 4.21E+06 |
| S5-7 | 0 | 1.37E+09 | 8.41E+06 |
| S6-1 | 7.65E+07 | 6.49E+08 | 2.27E+07 |
| S6-2 | 2.39E+07 | 3.84E+08 | 7.27E+06 |
| S6-3 | 3.38E+07 | 1.19E+09 | 7.79E+06 |
| S6-4 | 7.79E+06 | 3.58E+08 | 2.60E+06 |
| S6-5 | 1.48E+08 | 8.30E+08 | 4.67E+06 |
| S6-6 | 1.25E+07 | 8.62E+08 | 1.40E+07 |
| S6-7 | 1.56E+06 | 4.31E+08 | 6.23E+06 |
| S6-8 | 7.79E+05 | 2.77E+08 | 2.34E+06 |
| S6-9 | 7.79E+05 | 2.18E+08 | 0 |
| S7-1 | 6.83E+06 | 9.24E+07 | 3.60E+05 |
| S7-2 | 2.46E+07 | 7.35E+07 | 0 |
| S7-3 | 3.60E+07 | 2.71E+08 | 1.40E+06 |
| S7-4 | 2.90E+07 | 5.80E+08 | 9.35E+05 |
| S7-5 | 2.34E+06 | 3.52E+08 | 1.75E+06 |
| S7-6 | 2.33E+05 | 3.00E+07 | 1.86E+05 |
| S8-1 | 1.75E+08 | 2.41E+09 | 5.45E+07 |
| S8-2 | 4.60E+08 | 6.54E+09 | 5.19E+07 |
| S8-3 | 2.03E+07 | 1.10E+09 | 3.12E+06 |
| S8-4 | 9.35E+06 | 6.43E+08 | 3.12E+06 |
| S8-5 | 3.12E+07 | 8.94E+08 | 0 |
| S8-6 | 2.34E+07 | 7.96E+08 | 1.56E+06 |
| S8-7 | 1.66E+07 | 3.35E+08 | 9.35E+06 |
| S8-8 | 2.49E+07 | 7.56E+08 | 4.67E+06 |
| S8-9 | 4.99E+07 | 8.04E+08 | 7.79E+06 |
| S8-10 | 7.82E+05 | 3.04E+07 | 2.23E+05 |
| S8-11 | 2.92E+05 | 1.98E+07 | 0 |
| S8-12 | 5.84E+05 | 2.80E+07 | 3.89E+05 |
| S8-13 | 6.23E+05 | 1.51E+08 | 1.25E+06 |
| S8-14 | 2.79E+04 | 6.36E+06 | 0 |

**Reference:**

1 Passy, S. I. Spatial paradigms of lotic diatom distribution: a landscape ecology perspective. *J. Phycol.* **37**, 370-378 (2001).

2 Potapova, M. G. & Charles, D. F. Benthic diatoms in USA rivers: distributions along spatial and environmental gradients. *J. Biogeogr.* **29**, 167-187 (2002).

3 Soininen, J. Assessing the current related heterogeneity and diversity patterns of benthic diatom communities in a turbid and a clear water river. *Aquat. Ecol.* **38**, 495-501 (2004).

4 Soininen, J. & Weckström, J. Diatom community structure along environmental and spatial gradients in lakes and streams. *Fund. Appl. Limnol. /Archiv für Hydrobiologie* **174**, 205-213 (2009).

5 Heino, J. *et al.* Geographical patterns of micro‐organismal community structure: are diatoms ubiquitously distributed across boreal streams? *Oikos* **119**, 129-137 (2010).

6 Smucker, N. J. & Vis, M. L. Spatial factors contribute to benthic diatom structure in streams across spatial scales: considerations for biomonitoring. *Ecol. Indic.* **11**, 1191-1203 (2011).

7 Wang, J. *et al.* Patterns of elevational beta diversity in micro- and macroorganisms. *Global Ecol. Biogeogr.* **21**, 743-750 (2012).

8 Göthe, E., Angeler, D. G., Gottschalk, S., Löfgren, S. & Sandin, L. The influence of environmental, biotic and spatial factors on diatom metacommunity structure in Swedish headwater streams. *PloS One* **8**, e72237 (2013).

9 Tang, T., Wu, N., Li, F., Fu, X. & Cai, Q. Disentangling the roles of spatial and environmental variables in shaping benthic algal assemblages in rivers of central and northern China. *Aquat. Ecol.* **47**, 453-466 (2013).

10 Wu, N., Cai, Q. & Fohrer, N. Contribution of microspatial factors to benthic diatom communities. *Hydrobiologia* **732**, 49-60 (2014).

11 Passy, S. I. Diatom ecological guilds display distinct and predictable behavior along nutrient and disturbance gradients in running waters. *Aquat. Bot.* **86**, 171-178 (2007).
